# Interleukin-1 regulates follicular T cells during the germinal center reaction

**DOI:** 10.3389/fimmu.2024.1393096

**Published:** 2024-05-24

**Authors:** Aude Belbezier, Paul Engeroff, Gwladys Fourcade, Hélène Vantomme, Romain Vaineau, Bruno Gouritin, Bertrand Bellier, Isabelle Brocheriou, Nicolas Tchitchek, Stephanie Graff-Dubois, David Klatzmann

**Affiliations:** ^1^Sorbonne Université, INSERM, Immunology-Immunopathology-Immunotherapy (i3), Paris, France; ^2^Assistance Publique - Hôpitaux de Paris (AP-HP), Hôpital Pitié-Salpêtrière, Biotherapy (CIC-BTi) and Inflammation-Immunopathology-Biotherapy Department (i2B), Paris, France; ^3^Assistance Publique - Hôpitaux de Pari (AP-HP), Hôpital Pitié-Salpêtrière, Department of Pathology, Paris, France

**Keywords:** adaptive immunity, humoral response, vaccination, autoimmune diseases, immunotherapy

## Abstract

**Introduction:**

Antibody production and the generation of memory B cells are regulated by T follicular helper (Tfh) and T follicular regulatory (Tfr) cells in germinal centers. However, the precise role of Tfr cells in controlling antibody production is still unclear. We have previously shown that both Tfh and Tfr cells express the IL-1R1 agonist receptor, whereas only Tfr cells express the IL-1R2 decoy and IL-1Ra antagonist receptors. We aimed to investigate the role of IL-1 receptors in the regulation of B cell responses by Tfh and Tfr.

**Methods:**

We generated mice with IL-1 receptors inactivated in Tfh or Tfr and measured antibody production and cell activation after immunisation.

**Results:**

While IL-1β levels are increased in the draining lymph node after immunisation, antigen-specific antibody levels and cell phenotypes indicated that IL-1β can activate both Tfh and Tfr cells through IL-1R1 stimulation. Surprisingly, expression of IL-1R2 and IL-1Ra on Tfr cells does not block IL-1 activation of Tfh cells, but rather prevents IL-1/IL-1R1-mediated early activation of Tfr cells. IL-1Rs also regulate the antibody response to autoantigens and its associated pathophysiology in an experimental lupus model.

**Discussion:**

Collectively, our results show that IL-1 inhibitory receptors expressed by Tfr cells prevent their own activation and suppressive function, thus licensing IL-1-mediated activation of Tfh cells after immunisation. Further mechanistic studies should unravel these complex interactions between IL-1β and follicular helper and regulatory T cells and provide new avenues for therapeutic intervention.

## Introduction

Interleukin-1α and β (IL-1) are mainly known as potent proinflammatory cytokines in innate immunity (1–3). IL-1α is predominantly produced by pyroptotic cells and cells undergoing specific inflammasome-independent forms of cell death (4), while IL-1β is produced by hematopoietic cells such as blood monocytes and tissue macrophages in inflammatory conditions (3). All functions triggered by IL-1α and β are mediated through the agonist IL-1 receptor IL-1R1 (5). This interaction is tightly regulated by two other IL-1 receptors: (i) IL-1Ra, a soluble IL-1 antagonist receptor that binds to IL-1R1 and prevents the binding of IL-1 α/β and (ii) IL-1R2, a decoy of the IL-1 receptor located mainly in the membrane, which, when binding to IL-1, does not induce signal transduction, unlike the IL-1R1 receptor, thus reducing IL-1 availability (3).

Activated B cells undergo mutation, selection and affinity maturation in germinal centers (GCs) which leads to antibody (Ab) production by plasma cells and the generation of long-term memory B-cells. GC formation can occur independently of T cells or in T-cell dependent manner (6). Tfh cell derived CD40L expression, IL-4 and IL-21 play essential roles in GC B cell proliferation, survival and affinity maturation (7). The origin and mode of action of Tfr cells are more controversial. They are thought to derive from regulatory T cells (Tregs) and to act by regulating the help Tfh cell give to B-cells (8–12).

Recent work has suggested that IL-1 could be implicated in the T follicular helper cell (Tfh) and T follicular regulatory cell (Tfr) regulation of B cell immune responses (13, 14). Tfh cells express the IL-1R1 agonist receptor while Tfr cells also express IL-1R1, albeit at lower levels than Tfh cells, but also high levels of the IL-1R2 decoy receptor and IL-1Ra, the IL-1 receptor antagonist (13). Furthermore, antibody production is (i) reduced in IL-1–deficient mice (15, 16) and (ii) enhanced by adding IL-1 during immunization (17–19) or in mice lacking the expression of IL-1Ra (15, 16).

To investigate the role of each IL-1 receptor in Tfh and Tfr cell balance and antibody production, we generated mice specifically lacking IL-1 receptors in Tfh and/or Tfr cells and assessed B cell response after immunization.

## Material and methods

### Study design

For flow cytometry assays, the sample sizes were of at least three individuals per experiment. For enzyme-linked immunosorbent assay (ELISA), sample sizes were of at least three per condition. Sample size was determined on the basis of experimental feasibility and statistical significance. The experiments were not randomized. The investigators were not blinded to the allocation during experiments and analyses.

### Mice

B6.129S-IL1rntm1Dih/J (JAX stock #004754), B6.Cg-Tg(Cd4-cre)1Cwi/Bflu/J (JAX stock #022071), B6.129(Cg)-Il1r1tm1.1Rbl/J (JAX stock #028398), B6.129(Cg)-Foxp3tm4(YFP/icre)Ayr/J (JAX stock #016959) mice were provided by the Jackson Laboratory. C57BL/6N-Atm1BrdIl1r2tm1a(EUCOMM)Wtsi/WtsiPh (MGI:4842437) were provided by Institute of Molecular Genetics of the ASCR. Confirmed genotyping was performed in accordance with the supplier recommendations. All transgenic mice were in a C57BL/6J background. C57BL/6 Foxp3-GFP mice expressing GFP under the control of the promoter of Foxp3 gene were provided by B. Malissen of the Centre d’Immunologie de Marseille-Luminy (France). To generate CD4^cre^IL-1R1^lox^ mice, Il1r1tm1.1Rbl/J mice were mated with B6.Cg-Tg(Cd4-cre)1Cwi/Bflu/J mice. To generate FoxP3^creYFP^IL-1R1^lox^ mice Il1r1tm1.1Rbl/J mice were mated with B6.129(Cg)-Foxp3^tm4(YFP/icre)Ayr^/J mice. To generate Foxp3^GFP^CD4^cre^IL-1R1^lox^ mice, allowing to distinguish Tfh and Tfr cells, CD4^cre^IL-1R1^lox^ mice were crossed with Foxp3^GFP^ mice. To generate Foxp3^GFP^IL-1Ra^-/-^ mice, B6.129S-IL1rntm1Dih/J mice were crossed with Foxp3^GFP^ mice. To generate FoxP3^creYFP^IL-1R2^lox^ mice, C57BL/6N-A^tm1BrdI^l1r2^tm1a(EUCOMM)Wtsi/Wtsi^ mice were mated with B6.129(Cg)-Foxp3^tm4(YFP/icre)Ayr^/J mice. All animals were maintained at the Sorbonne University (SU), Centre d’Expérimentation Fonctionnelle animal facility (Paris, France) under specific pathogen–free conditions in agreement with current European legislation on animal care, housing, and scientific experimentation (agreement number A751315). All procedures were approved by the local animal ethics committee.

### Immunization models

#### OVA immunization models

Mice were either immunized with intraperitoneal injection three times (D0, D2, D4) and sacrificed at D8 for Tfr/Tfh cell transcriptomic and *in vitro* studies or immunized twice (D0 and D14). Intraperitoneal injection was performed with 100 ug of OVA (OVA A5503, Sigma-Aldrich) mixed with 500 µg of aluminum hydroxide (Alum) gel (AlH303, Sigma). Mice were also immunized with OVA-Alum subcutaneously (s.c), in the flank one time and sacrificed at D0, D3, D6, D9, D12, D15 for deep immunophenotyping of follicular cells or to measure the level of IL-1 from inguinal draining lymph.

#### Pristane immunization model

Lupus was induced by intraperitoneal injection with 100 µg of Pristane at D0. Mice were euthanized at 6 months (M6) for the quantification of immune complex deposition in the kidney.

### Cell sorting

Splenocytes from immunized mice were stained with Ter-119–biotin, CD8-biotin, CD11c-biotin and B220-biotin antibodies for 20 min at 4°C and labeled with anti-biotin magnetic beads (Miltenyi Biotec) for 15 min at 4°C. Biotinylated cells were depleted on an autoMACS separator (Miltenyi Biotec), following the manufacturer’s procedure. Enriched T cells were stained as described in the “Flow cytometry analysis of human cells” section, and the following subsets were sorted on BD FACSAria II (BD Biosciences), with a purity of >98%: CD4^+^CD8^−^CXCR5^hi^PD1^hi^Foxp3^−^ Tfh cells, CD4^+^CD8^−^CXCR5^hi^PD^hi^Foxp3^+^ Tfr cells. Inguinal lymph nodes were comminuted mechanically and filtered through a cell strainer (35 μm, StemCell). The diluent was stored at -80°C to perform IL-1β ELISA. The cell suspension was transferred into a 5 mL tube (Falcon) containing PBS, washed twice with PBS medium and stained.

### Analysis of lupus kidney involvement and research of immune complex deposition

One kidney/mouse was embedded in OCT Tissue-Tek compound (CellPath), snap-frozen, and stored at -80˚C until use. Kidneys were frozen with optimal cutting temperature (OCT) compound, cut into 7 μm sections and fixed with acetone. Sections will then be stained with FITC-conjugated anti-C3 and Alexa Fluor 647-conjugated anti-mouse IgG (Abcam, Cambridge, UK) antibodies. DNA was visualized using DAPI (Sigma-Aldrich). Quantification of fluorescence will be performed at ×20 magnification in one random microscopic field of each kidney, followed by binary analysis using Image J 1.49v software. Scores on a scale of 0–3 were estimated for both C3 and IgG deposition, based on the fluorescence intensity (0 = no deposits, 1 = low, 2 = moderate, 3 = high). An intensity threshold of 2 was considered positive for IgG or D3 deposits. Immune complex deposits positivity was assessed by a score above 2 for IgG and C3.

### Flow cytometry analysis

Fresh total cells from spleens were isolated in PBS1×–3% fetal bovine serum (FBS) and stained for 20 min at 4°C with the following monoclonal antibodies at predetermined optimal dilutions: CD121b-BV421, CD19-PeCF594, CD4-V500, CD8a-AF700, Bcl6-APC, CXCR5-Biotin, GL7- e450, CD95 PE, Foxp3-AF488, PD-1–PE (BD Biosciences) or PE-Texas Red (PETR), streptavidin-APC or streptavidin–APC-Cy7 (BD Biosciences), GITR PETR (Miltenyi). CXCR5 staining was performed using biotinylated anti-CXCR5 for 30 min at 20°C followed by APC- or APC-Cy7–labeled streptavidin at 4°C. Intracellular detection of Foxp3 was performed on fixed and permeabilized cells using appropriate buffer (eBioscience), following the manufacturer’s recommendations. Stained cells were run on CytoFLEX S cytometer (Beckman - Coulter) and analyzed using FlowJo software (TreeStar Inc.). Dead cells were excluded by forward/side scatter gating.

### Spectral flowx cytometry profiling

Two million cells per sample were stained. Cell viability was assessed by Viability UV T (Thermofisher). Cells were washed with PBS. After washing, anti-chemokine antibody (CXCR5 (BD Biosciences)) and brilliant stain buffer (used in complement of Brilliant fluorescent polymer dyes) were added in PBS 1 × at room temperature for 20 minutes. Other membrane markers were added next (CD3 (Biolegend), CD4 (Biolegend), CD8 (Biolegend) CD11b (BD Biosciences), PD1 (BD Biosciences), ICOS (BioLegend), Fas (BD Biosciences), GL7 (BioLegend), CTLA4 (BD Biosciences), GITR (BD Biosciences), CD25 (BD Biosciences), IgD (BD Biosciences), IgG (BD Biosciences), CD38 (BD Biosciences), B220 (Biolegend), CD19 (Biolegend)) and lymph node cells were incubated for 30 additional minutes at room temperature. After washing, the cells were washed and then resuspended in a residual volume of 100uL and acquired on an AURORA. Supervised analyses, conducted using FlowJo software, were used to study the phenotype and function of some specific T and B cell populations (**Supplementary Figure S2**).

For unsupervised analysis, batch-corrected expression values for all markers and cell clusters analyses were performed using Imubac R package (20, 21). The FlowSOM-guided clustering initially identified 40 clusters. Clusters were then manually analyzed and annotated. Five cell populations have been identified and annotated as follows: CD19^+^ (CD19^+^CD4^-^CD8^-^CD11b^-^), CD4^+^ (CD19^-^CD4^+^CD8^-^CD11b^-^), CD8^+^ (CD19^-^CD4^-^CD8^+^CD11b^-^), and CD11b^+^ (CD19^-^CD4^-^CD8^-^CD11b^+^). Additionally, 9 cell subpopulations have been identified and annotated as follows: CD8^+^, CD11b^+^, Tfr (CD4^+^CD8^-^CD19^-^CD11b^-^Foxp3^+^CXCR5^+^PD1^+^), Treg (CD4^+^CD8^-^CD19^-^CD11b^-^Foxp3^+^CXCR5^-^PD1^-^), Tfh (CD4^+^CD8^-^CD19^-^CD11b^-^Foxp3^-^CXCR5^+^PD1^+^), Tconv (CD4^+^CD8^-^CD19^-^CD11b^-^Foxp3^+^CXCR5^-^PD1^-^), Plasmablast (CD19^+^CD38^+^B220^low^IgG^+^IgD^-^), GCB (CD19^+^GL7^+^Fas^+^IgD^-^), and Transitional B cell (CD19^+^CD38^-/low^B220^low^IgD^-^GL7^+^Fas^+^). A cell count for each cell population and subpopulation was obtained for each sample. The proportion of each cell subpopulation was then obtained by computing the proportions of cell subpopulations among cell population.

### Gene expression analysis based on RT-PCR

Sorted cells were washed in PBS1× and stored in RNAqueous kit lysis buffer (Ambion Inc./Life Technologies) at -80°C. Total RNA was extracted according to the manufacturer’s instructions, and quality was assessed on a bioanalyzer using the Pico RNA Reagent Kit (Agilent Technologies). For quantitative PCR, we analyzed 25 ng of cDNA for expression of a range of genes using the GeneAmp 5700 Sequence Detection System (Applied Biosystems). The samples were normalized to the GADPH protein as control mRNA, by change in cycling threshold (ΔCT) method and calculated based on 2-ΔΔCT.

### *In vitro* assay

Tfr cells were sorted from D-8 OVA-immunized mice, following a previously described “Immunization” protocol. For all the conditions, 1 × 10^4^ Tfr cells were cultured for 12 hours in 96-well plates (Nunc) in complete RPMI 1640 (Thermo Scientific) and three CD3/CD28 beads for one T cell (Dynabeads Mouse T- Activator, Thermo Scientific). We then added or not recombinant mouse 1 μg of IL-1β (Milteniy Biotec) to Tfr cell cultures. IL-10 production by Tfr cells was measured by ELISA (eBioscience) in supernatants of cultured Tfr cells. Expression of activation marker (GITR) was analyzed by flow cytometry.

### ELISA

The concentrations of mouse IL-10 (eBiosciences), IL-1Ra (eBiosciences), IL-1β (eBiosciences) in the culture supernatant, lymph node lavage fluid and serum were detected using ELISA kits. The serum levels of anti-OVA, anti-DNA and anti-RNP antibodies were detected with in-house ELISA. Briefly, 5 ng/ml OVA proteins were coated on 96-well high-binding EIA/RIA plates overnight. After blocking, mouse serum that had been diluted at different concentration (1 : 4000, 1:12000, 1: 36000, 1:108000, 1:324000, 1:972000) was added to the plates and incubated for 2 h; then HRP- conjugated anti-mouse IgG (Southern Biotech) was added at a 1 : 2500 dilution followed by Streptavidin revelation. For ELISA quantifying anti-DNA and anti-RNP antibodies, 200 µg/ml DNA or 1,33 µg/ml RNP proteins were coated on 96-well high-binding EIA/RIA plates overnight. After blocking, mouse serum that had been diluted at different concentration (1:100, 1:300, 1:900, 1:2700) was added to the plates and incubated for 2 h; then HRP- conjugated anti-mouse IgG (Southern Biotech) was added at a 1: 2500 dilution followed by Streptavidin revelation. For total IgG levels in serum, Maxisorp (Nunc) plates were coated with anti-mouse Ig (ref: 0107-01, SouthernBiotech) on 96-well high-binding EIA/RIA plates overnight. After blocking, mouse serum that had been diluted at different concentration was added to the plates and incubated for 2 h; then HRP- conjugated anti-mouse IgG (ref: 1030-01, Southern Biotech) was added at a 1: 2500 dilution followed by Streptavidin revelation.

### Statistical analysis

All statistical tests were performed by using GraphPad PRISM 6.0 (GraphPad Software, Inc, La Jolla, Calif). For all experiments throughout this article, a p-value threshold of 0.05 and statistical significance are displayed as ∗P less than or equal to.05; ∗∗P less than or equal to.01; ∗∗∗P less than or equal to.001; and ∗∗∗∗P less than or equal to.0001. Two groups were analyzed by non-parametric Mann-Whitney U test for unpaired data and paired Wilcoxon for paired data.

## Results

### IL-1 mediates Tfh and Tfr cell activation

**Figure 1:**
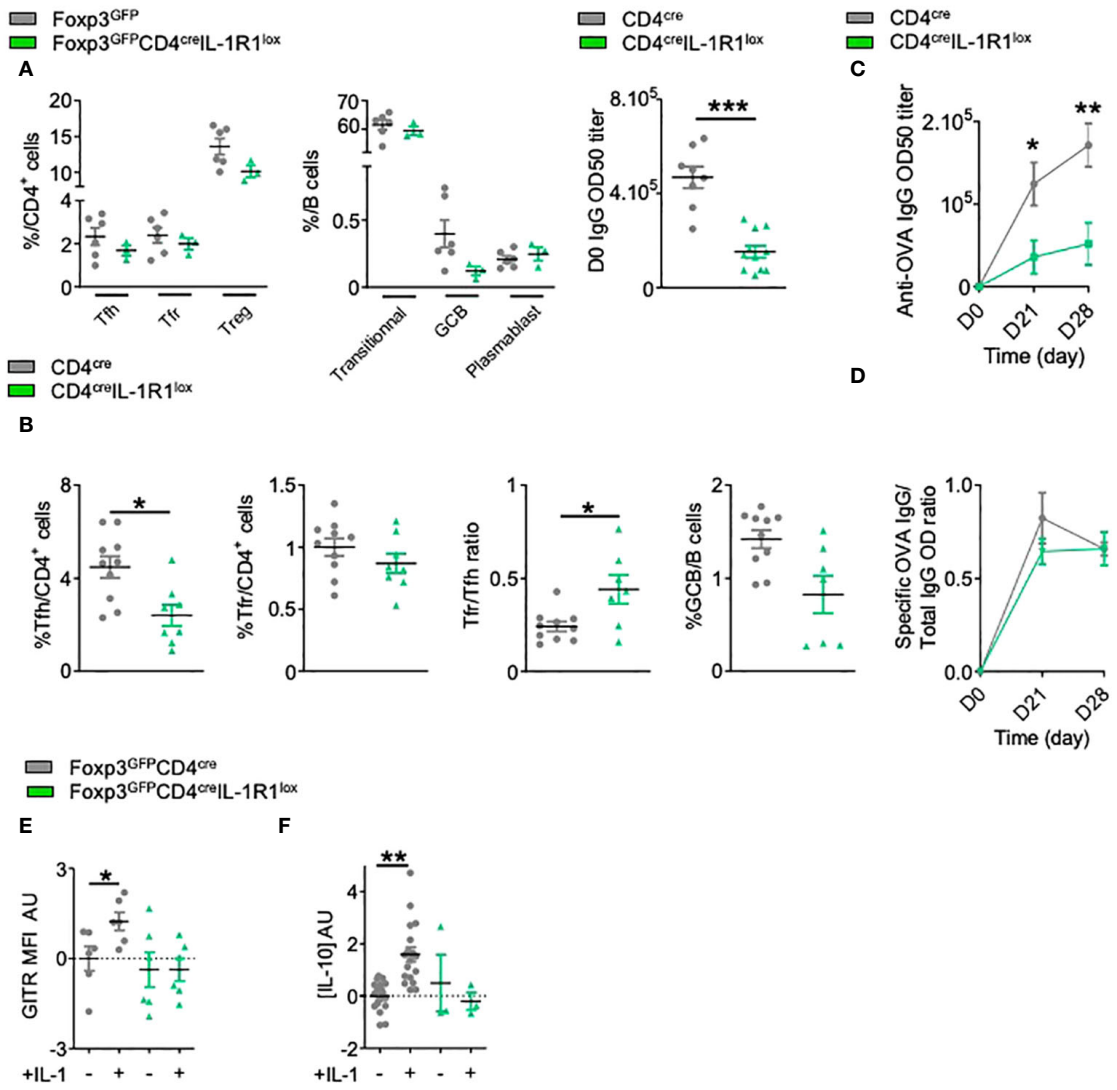
IL-1 regulates Tfh cell but also Tfr cell activation. **(A)** Spectral flow cytometry quantification of the percentages of CD4^+^ T and B cell populations within SLOs and circulating IgG antibody levels before any immunization in CD4^cre^IL-1R1^lox^ (green triangle) and CD4^cre^ (grey circle) mice. **(B)** Flow cytometry quantification of Tfh, Tfr, GCB cells and Tfr/Tfh ratio 28 days after intraperitoneal immunization with OVA-Alum in CD4^cre^IL-1R1^lox^ (green triangle) and CD4^cre^ mice (grey circle). **(C)** Circulating anti-OVA IgG antibody level after intraperitoneal immunization with OVA-Alum in CD4^cre^IL-1R1^lox^ (green triangle) and CD4^cre^ (grey circle) mice from D0 to D28. **(D)** Ratio of specific anti-OVA IgG Ab/total IgG Ab after OVA immunization in CD4^cre^IL-1R1^lox^ (green triangle) and CD4^cre^ (grey circle) mice from D0 to D28. **(E, F)** Histograms comparing the MFI of GITR **(E)** and the level of IL-10 production **(F)** of Tfr cells from Foxp3^GFP^CD4^cre^IL-1R1^lox^ (green triangle) and Foxp3^GFP^ (grey circle) mice cultured with or without IL-1. GITR MFI and IL-10 levels are expressed in arbitrary units that correspond to data centered and reduced relative to Tfr from Foxp3^GFP^ (grey) mice grown without IL-1. *P < 0.05, **P < 0.01, ***P < 0.005, Mann-Whitney U test for unpaired data and Wilcoxon paired test for paired data. **(B–F)** Data are representative of three independent experiments.

In CD4^+^ T cells, IL-1R1 is mostly expressed by Tfh cells and at a lower level on Tfr cells (15). We crossed CD4^cre^ mice with IL-1R1^lox^ mice and observed a proper deletion of IL-1R1 on CD4^+^ cells (**Supplementary Figure S1A**). At steady state, CD4^cre^IL-1R1^lox^ mice had normal numbers of Tfh, Tfr or Treg cells, and normal numbers of transitional B cells (CD19^+^CD38^-/low^B220^low^IgD^-^GL7^-^), GC B cells (GCBs) (CD19^+^GL7^+^Fas^+^IgD^-^) or plasmablasts (CD19^+^CD38^+^B220^low^IgG^+^IgD^-^). However, they had significantly reduced serum IgG Ab levels (**Figure 1A**). Four weeks after their immunization with ovalbumin (OVA), we observed a significant reduction of the proportion of Tfh but not of Tfr cells, leading to a significantly increased Tfr/Tfh ratio in comparison with control mice (**Figure 1B**). This was accompanied by a markedly decreased production of GCBs (CD19^+^GL7^+^Bcl6^+^IgD^-^) (**Figure 1B**). These effects translated into a significant decrease of anti-OVA Ab production (**Figure 1C**), whereas the ratio of OVA-specific Ab to total Ab was not affected (**Figure 1D**). These results indicate an effect of IL-1R1 on the Tfh/Tfr control of humoral responses.

We confirmed the functionality of IL-1R1 on Tfr cells *in vitro*. The addition of IL-1β to purified Tfr from OVA-immunized mice significantly increased IL-10 production and GITR expression (**Figures 1E, F**), and such activation could no longer be detected with IL-1R1-deleted Tfr (**Figures 1E, F**). Collectively, these results suggest a functional role of IL-1R1 on Tfr cells, which calls for study of the impact of specific depletion of IL-1R1 on Tfr cells.

**Figure 2:**
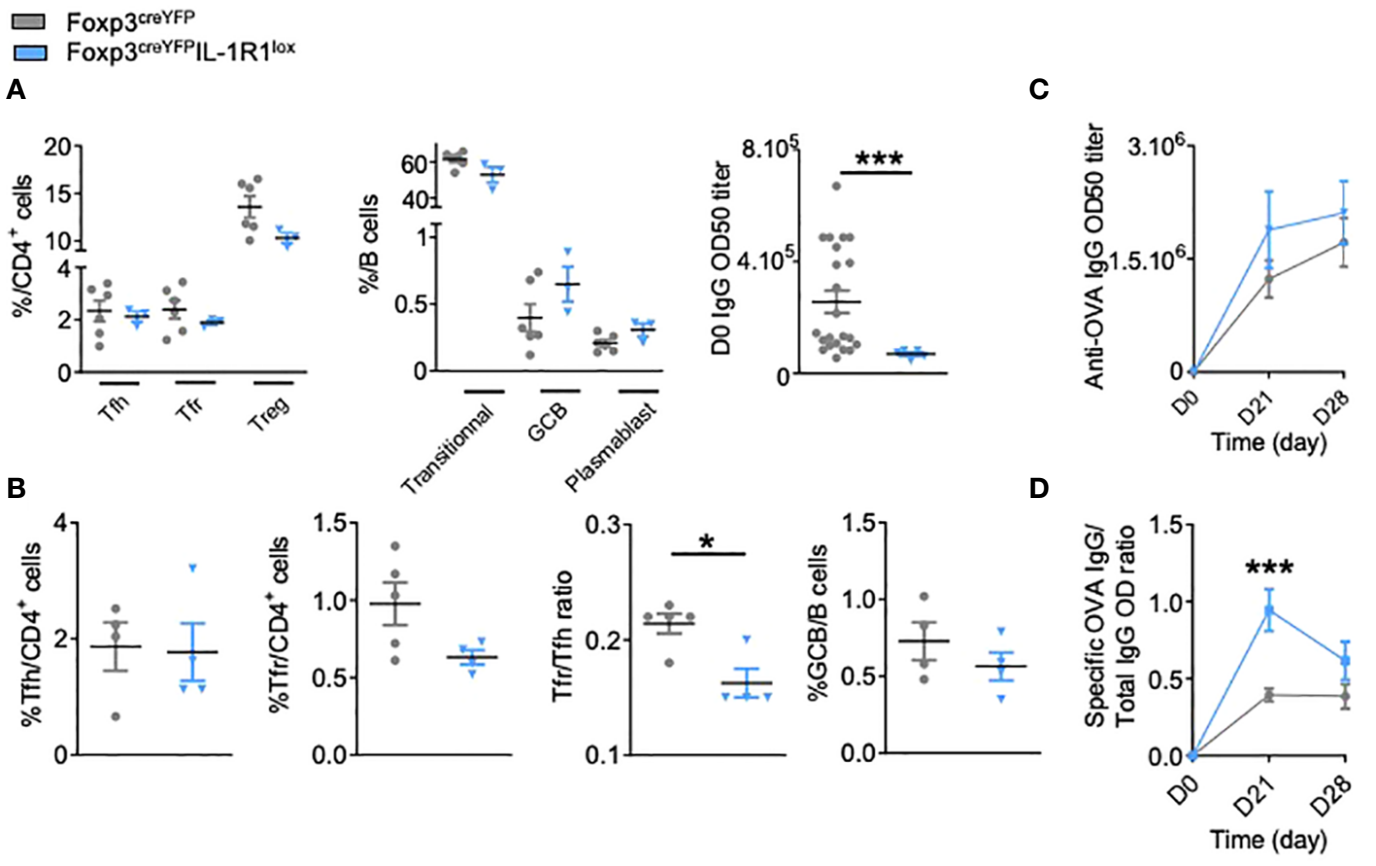
IL-1R1 receptor expressed by Tfr cells is functional and critical for the regulation of the humoral immune response. **(A)** Spectral flow cytometry quantification of CD4^+^ T and B cell populations within SLOs and circulating IgG antibody levels before any immunization in Foxp3^creYFP^IL-1R1^lox^ (blue triangle) and Foxp3^creYFP^ mice (grey circle). **(B)** Flow cytometry quantification of Tfh, Tfr, GCB cells and Tfr/Tfh ratio 28 days after intraperitoneal immunization with OVA-Alum in Foxp3^creYFP^IL-1R1^lox^ (blue triangle) and Foxp3^creYFP^ mice (grey circle). **(C)** Circulating anti-OVA IgG antibody level after intraperitoneal immunization with OVA-Alum in Foxp3^creYFP^IL-1R1^lox^ (blue triangle) and Foxp3^creYFP^ mice (grey circle) from D0 to D28. **(D)** Ratio of specific anti-OVA IgG Ab/total IgG Ab after OVA immunization in Foxp3^creYFP^IL-1R1^lox^ (blue triangle) and Foxp3^creYFP^ mice (grey circle) at the times indicated. *P < 0.05, ***P < 0.005, Mann-Whitney U test. **(B–D)** Data are representative of three independent experiments.

We thus crossed Foxp3^creYFP^ mice with IL-1R1^lox^ mice. We confirmed that Foxp3^creYFP^IL-1R1^lox^ mice had a proper deletion of IL-1R1 expression on Tfr but not on Tfh cells (**Supplementary Figure S1B**). At steady state, compared to control mice, Foxp3^creYFP^IL-1R1^lox^ mice presented (i) no significant modifications of Tfh, Tfr or Treg cells, nor the B cell subsets (**Figure 2A**) and (ii) a significantly lower level of circulating IgG Ab (**Figure 2A**). Four weeks after immunization, compared to controls, they had a significantly decreased Tfr/Tfh ratio (**Figure 2B**) and an increase of specific anti-OVA Ab (**Figure 2C**), with a significantly increased ratio of specific to total Ab (**Figure 2D**). It should be noted that the proportion of Treg in CD4^cre^IL-1R1^lox^ and Foxp3^creYFP^IL-1R1^lox^ mice is not changed after immunization (**Supplementary Figure S3**), which supports the idea that IL-1R1 deletion mainly affects Tfr cells. Altogether, these results indicate that the expression of IL-1R1 on Tfr cells is involved in their own activation.

### IL-1R2 and IL-1Ra control humoral immunity

**Figure 3:**
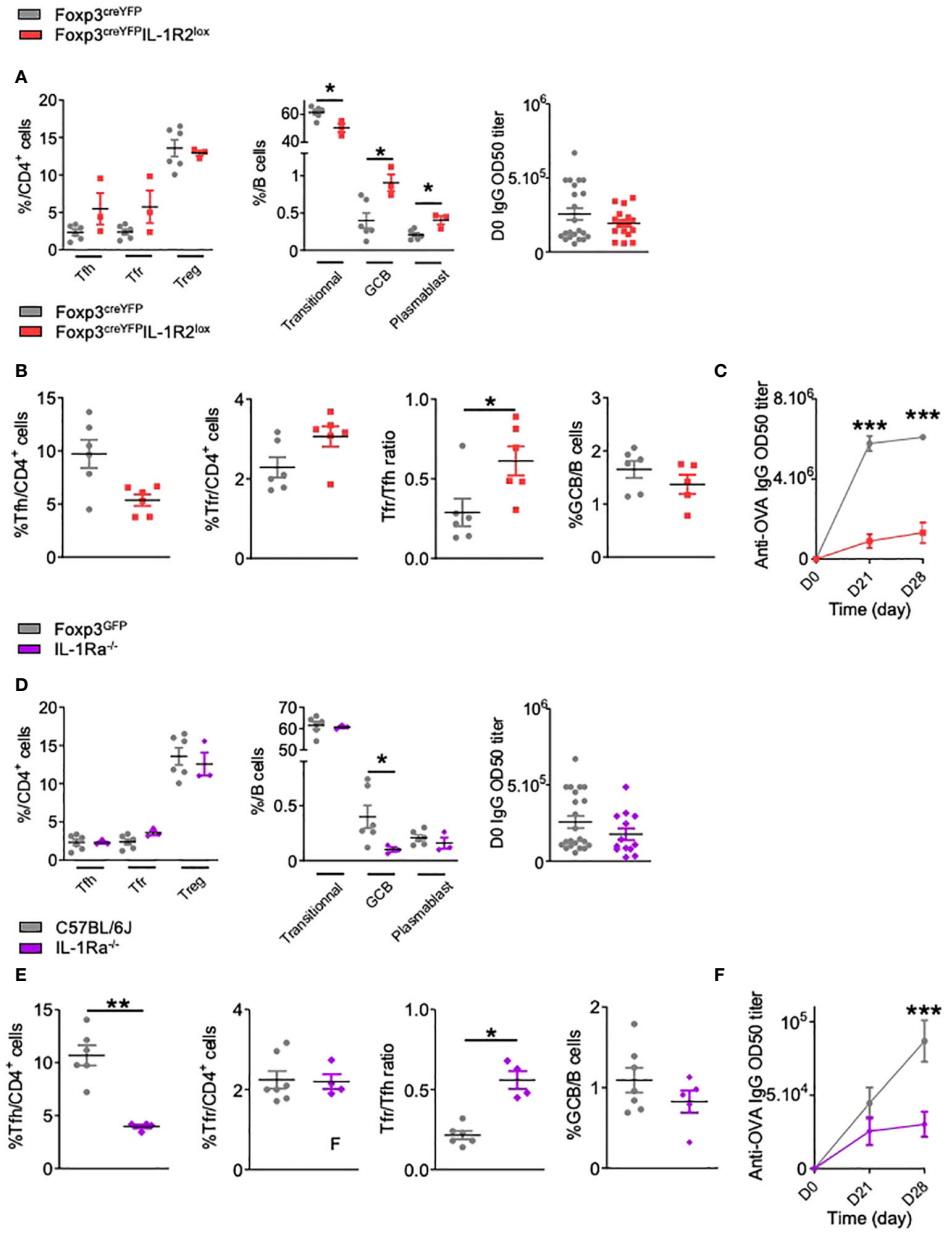
IL-1R2 and IL-1Ra antagonist receptor are functional and critical for the regulation of the humoral immune response. **(A)** Spectral flow cytometry quantification of CD4^+^ T and B cell populations within SLOs and circulating IgG antibody levels before any immunization in Foxp3^creYFP^IL-1R2^lox^ (red square) and Foxp3^creYFP^ (grey circle) mice. **(B)** Flow cytometry quantification of Tfh, Tfr, GCB cells and Tfr/Tfh ratio 28 days after intraperitoneal immunization with OVA-Alum and **(C)** circulating anti-OVA IgG antibody level after intraperitoneal immunization with OVA-Alum in Foxp3^creYFP^IL-1R2^lox^ (red square) and Foxp3^creYFP^ (grey circle) mice from D0 to D28. **(D)** Quantification of CD4^+^ T, B cell subsets within SLOs and circulating IgG antibody levels before any immunization in Foxp3^GFP^IL-1Ra^-/-^ (purple diamond) and Foxp3^GFP^IL-1ra^WT^ (grey circle) mice. **(E)** Proportion of Tfh, Tfr, GCB cells and Tfr/Tfh ratio 28 days after intraperitoneal immunization with OVA-Alum and **(F)** circulating anti-OVA IgG antibody level after intraperitoneal immunization with OVA-Alum in IL-1Ra^-/-^ (purple diamond) and Foxp3^GFP^ (grey circle) mice. *P < 0.05, **P < 0.01, ***P < 0.005, Mann-Whitney U test. **(B, C, E, F)** Data are representative of three independent experiments.

To investigate the role of IL-1R2 in Tfr cell activation, we crossed Foxp3^creYFP^ mice with IL-1R2^lox^ mice. At steady state, we observed a non-significant increase of follicular T cells including both Tfh and Tfr in Foxp3^creYFP^IL-1R2^lox^ mice compared to control mice (**Figure 3A**). However, Foxp3^creYFP^IL-1R2^lox^ mice showed a significant decrease in the proportion of transitional B cells, with a significant increase in the proportion of GCBs and plasmablasts (**Figure 3A**). This suggests that deletion of IL-1R2 on Tfr specifically impacts B cell maturation, even though circulating IgGs were not affected in naïve mice (**Figure 3A**). Four weeks after OVA-immunization, we observed a significant increase of the Tfr/Tfh ratio in FoxP3^creYFP^IL-1R2^lox^ mice compared to control mice, whereas the GCB/B ratio remained similar (**Figure 3B**). Interestingly, FoxP3^creYFP^IL-1R2^lox^ mice produced greatly reduced levels of OVA-specific IgG compared to control mice (**Figure 3C**).

The role of IL-1Ra on Tfr cells was investigated in IL-1Ra^-/-^ mice in the absence of a direct means of eliminating IL-1Ra only in Tfr cells (**Figures 3D-F**). At steady state, IL-1Ra deletion did not affect the proportion of Tfh and Tfr cells (**Figure 3D**). In addition, circulating IgG were not affected, whereas GCB cells were significantly decreased (**Figure 3D**). Four weeks after OVA-immunization, and similarly to Foxp3^creYFP^IL-1R2^lox^ mice, we observed a significant decrease in OVA-specific IgG production, whereas the proportion of GCBs was not significantly altered (**Figures 3E, F**) Of note, the proportion of Treg in Foxp3^creYFP^IL-1R2^lox^ and Il-1Ra^-/-^ mice is not altered after immunization (**Supplementary Figure S3**), suggesting that IL-1R2 and IL-1Ra deletion mainly affects Tfr cells. Overall, our data indicate that IL-1R2 and IL-1Ra on Tfr cells are critical for the production of antigen-specific antibodies.

### IL-1R2 and IL-1Ra antagonist receptor prevent IL-1β-mediated activation of Tfr cells

**Figure 4:**
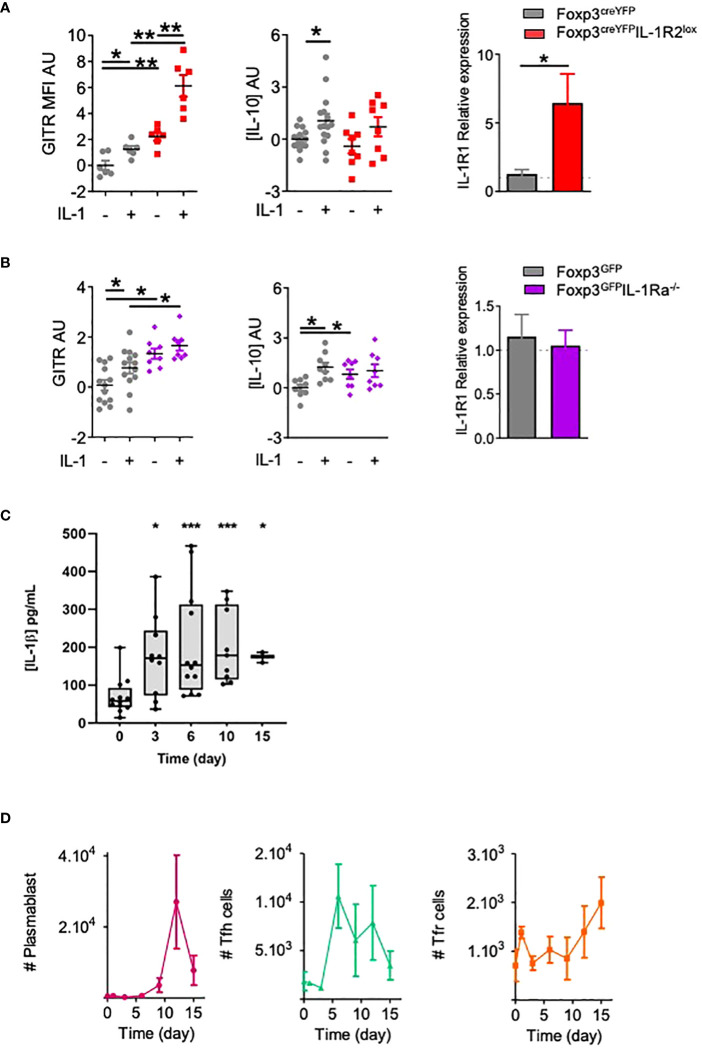
IL-1R2 and IL-1Ra inhibitory receptors prevent IL-1β-mediated activation of Tfr cells. Quantification of GITR expression, IL-10 production and IL-1R1 expression by Tfr cells from **(A)** Foxp3^creYFP^IL-1R2^lox^ (red square) and Foxp3^creYFP^ **(B)** Foxp3^GFP^IL-1Ra^-/-^ mice (purple diamond) and Foxp3^GFP^ mice **(B)** (grey circle), activated with or without IL-1. GITR MFI and IL-10 levels are expressed in arbitrary units that correspond to data centered and reduced relative to Tfr from respective control mice cultivated without IL-1. IL-1R1 expression is evaluated as the relative expression corresponding to the IL-1R1 expression level of the Tfr cell population compared to its relative level in control mice. **(C, D)** Kinetics of IL-1β production (grey) **(C)**, plasmablasts (pink), Tfh cells (green) and Tfr cells (orange) **(D)** in draining lymph nodes from C57BL/6 mice at D0, D3, D6, D9, D12 and D15 after subcutaneous Alum-OVA immunization. Data represent the median of 3 mice ± SEM. *P < 0.05, **P < 0.01, ***P < 0.005, Mann-Whitney U test for unpaired data and Wilcoxon paired test for paired data. **(A, B)** Data are representative of three independent experiments. #: number of indicated cells.

To go further into the characterization of IL-1R2 and IL-1Ra in Tfr cell functions, we developed functional assays where Tfr cells were cultured in the presence of IL-1β. Compared with control Tfr cells, Tfr cells derived from Foxp3^creYFP^IL-1R2^lox^ or Foxp3^GFP^IL-1Ra^-/-^ mice showed increased expression of GITR without IL-1β stimulation (**Figures 4A, B**). Upon IL-1β stimulation, expression of GITR was even more enhanced in Tfr from Foxp3^creYFP^IL-1R2^lox^ mice, whereas it remained stable in Tfr from Foxp3^GFP^IL-1Ra^-/-^ mice. Of note, in the absence of IL-1β, IL-1Ra-deleted Tfr cells produced more IL-10 than control Tfr cells (**Figure 4B**), whereas production of IL-10 by Tfr from Foxp3^creYFP^IL-1R2^lox^ mice was equivalent to controls (**Figure 4A**). IL-10 production was enhanced by the addition of IL-1β in control Tfr cells, whereas it did not affect IL-10 production by Tfr from Foxp3^creYFP^IL-1R2^lox^ and Foxp3^GFP^IL-1Ra^-/-^ mice. Noteworthily, IL-1R2 but not IL-1Ra deletion on Tfr cells enhanced their expression of IL-1R1, suggesting a feedback loop of regulation in between IL-1R2 and IL-1R1 (**Figures 4A, B**).

Finally, we assessed the dynamics of IL-1β concentrations in the draining lymph nodes of OVA-immunized mice. IL-1β was detected as early as day 0, but its concentration peaked between D6 and D10 (**Figure 4C**). This increase preceded the proliferation of Tfr, which appeared after D9 (**Figure 4D**). Based on these results, we propose that IL-1 inhibitory receptors are expressed by Tfr to prevent their early own activation by IL-1, which is present at low levels early after immunization.

### Tfr IL-1R expression regulates auto-antibody response in experimental lupus

We then evaluated whether this control of humoral immunity could also affect the autoimmune humoral response. We used pristane-induced lupus in which pristane injection induces mainly anti-RNP antibodies in C57BL/6 mice (22), but also anti-DNA antibodies and renal lesions with glomerular immune complex deposits at 6 months (22, 23).

**Figure 5:**
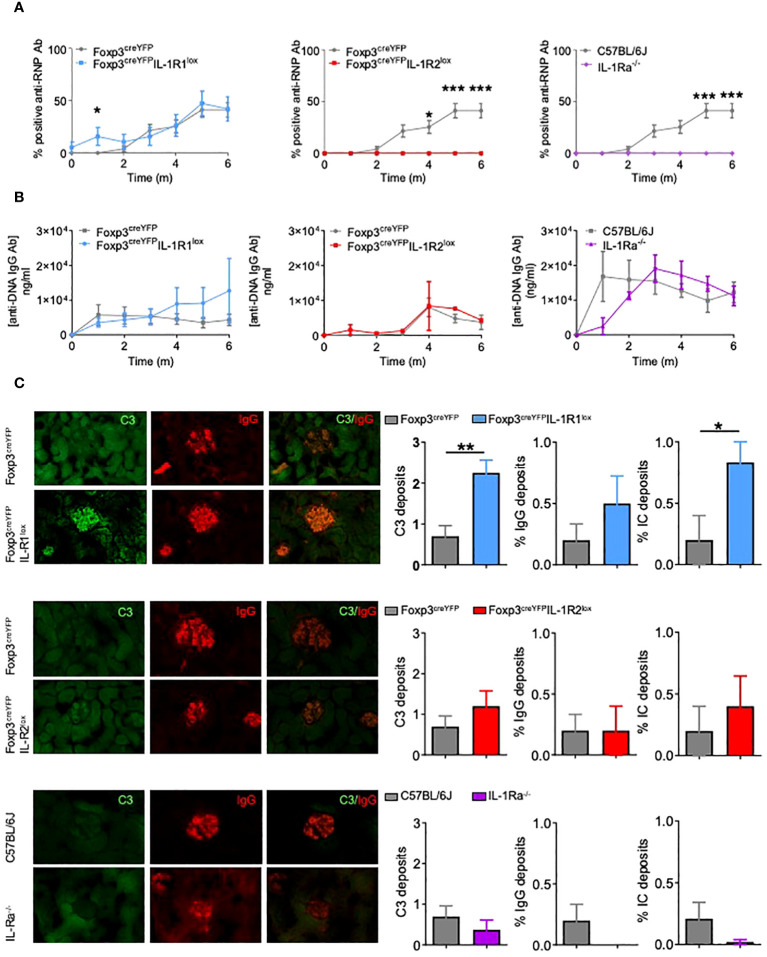
Deletion of IL-1R1 on Tfr cells does not alter the risk of autoimmunity, but deletion of IL-1R2 and IL-1Ra on Tfr cells prevents autoimmune disease. **(A)** Proportion of mice with positive anti-RNP Ab after pristane immunization in Foxp3^creYFP^IL-1R1^lox^ (blue, n= 6), Foxp3^creYFP^IL-1R2^lox^ (red, n= 6), IL-1Ra^-/-^ (purple, n= 6) and control (grey, Foxp3^YFP^, Foxp3^YFP^ or and Foxp3^GFP^ respectively, n= 6) mice. **(B)** Circulating anti-DNA IgG antibody level after pristane immunization in Foxp3^creYFP^IL-1R1^lox^ (blue, n= 6), Foxp3^creYFP^IL-1R2^lox^ (red, n= 6), IL-1Ra^-/-^ (purple, n= 6) and control (grey, Foxp3^YFP^, Foxp3^YFP^ or and Foxp3^GFP^ respectively, n= 6) mice. **(C)** Deposition of C3 (green) and IgG (red) in glomeruli of kidney tissues from Foxp3^creYFP^IL-1R1^lox^ (blue, n=6), Foxp3^creYFP^IL-1R2^lox^ (red, n=5), IL-1Ra^-/-^ (purple, n=5) and control (grey, Foxp3^creYFP^and Foxp3^GFP^, n=10) mice exposed to pristane. Intensity of C3 deposition is evaluated using a scale ranging from 0: absent, 1: weak, 2: moderate to 3: intense and IgG deposition is defined as an IgG immunofluorescence score greater than 2. The proportion of mice with immune complex deposition is defined as IgG and C3 immunofluorescence score greater than 2 in Foxp3^creYFP^IL-1R1^lox^ (blue), Foxp3^creYFP^IL-1R2^lox^ (red), IL-1Ra^-/-^ (purple) and control (grey, Foxp3^YFP^ and Foxp3^GFP^) mice after 6 months of exposure to pristane. *P < 0.05, **P < 0.01, ***P < 0.005, Mann-Whitney U test. **(A, B)** Data are representative of three independent experiments. .

Remarkably, while both Foxp3^creYFP^IL-1R1^lox^ mice and control mice developed anti-RNP antibodies (**Figure 5A**), Foxp3^creYFP^IL-1R2^lox^ and IL-1Ra^-/-^ mice did not (**Figure 5A**). In contrast, the level of anti-DNA antibodies produced by control and mutant mice was similar (**Figure 5B**).

Foxp3^creYFP^IL-1R1^lox^ mice showed more glomerular C3 and immune complex depositions than control mice, while the proportion of glomeruli affected by IgG deposition was not significantly increased (**Figure 5C**). Foxp3^creYFP^IL-1R2^lox^ mice showed similar rates of C3, IgG, or immune complex deposition as controls (**Figure 5B**), despite the absence of anti-RNP production. Similarly, IL-1Ra^-/-^ mice showed a non-significant decrease of immune complex deposition. Altogether, results indicate that deletion of IL-1R2 and IL-1Ra on Tfr abolishes the production of anti-RNP antibodies, whereas IL-R1 deletion on Tfr enhances renal damage.

## Discussion

Our study sheds light on the intricate roles of IL-1β in orchestrating the balance between Tfh and Tfr cells, crucial for fine-tuning antibody responses. Notably, the modulation of Tfh and Tfr functions via IL-1β signaling presents a complex mechanism that diverges from and adds to the current understanding of immune regulation. Echoing the findings from earlier research by our team, we recognize the critical role of IL-1 in regulating the balance between Tfh and Tfr cells, specifically highlighting IL-1’s importance in controlling humoral responses, thereby supporting the potential therapeutic implications of modulating IL-1β signaling pathways in autoimmune diseases or allergies (14). Previous research has underscored the importance of Tfh cells in supporting B cell maturation and antibody production within germinal centers (24). In contrast, Tfr cells, as highlighted by our findings, serve a regulatory function, maintaining immune homeostasis and preventing autoimmunity (25). Our results align with these perspectives but further delineate the specific contributions of IL-1β signaling pathways to these processes.

Comparatively, our observations on the differential expression of IL-1R1, IL-1R2, and IL-1Ra on Tfr cells introduce a novel layer of complexity. In our experimental settings, the function of IL-1R on Tfh cannot be addressed specifically but can be inferred by comparing the phenotypes of CD4^cre^IL-1R1^lox^ mice and Foxp3^creYFP^IL-1R1^lox^ mice. The absence of IL-1R1 on Tfh and Tfr prevents Tfh expansion and decreases the expression of activation markers post-immunization. However, the lack of IL-1R1 on Tfr should reduce their activation and suppressive function on Tfh, and thus cannot be responsible for the reduced Tfh activation. Thus, according to the results obtained in these mice it can be concluded that the IL-1/IL-1R1 interaction contributes to Tfh activation and expansion triggered by immunization. Indeed, loss of IL-1R1 on CD4 T cells results in defective production of anti-OVA IgG after immunization. Moreover, at steady state, the absence of IL-1R1 on CD4 T cells translates into a reduction of the IgG titer, suggesting that IL-1R1 is required for IgG production. Finally, while we previously overlooked the low IL-1R1 expression by Tfr, we show here that this receptor is functional. Therefore, IL-1 could result in the activation of both Tfh and Tfr, which would have opposite functions.

While the existing literature has extensively documented the pro-inflammatory roles of IL-1β (26), our study uniquely identifies how IL-1β’s interaction with its receptors on Tfr cells intricately modulates the immune response, suggesting a more nuanced role than previously understood.

Furthermore, the impact of IL-1R1, IL-1R2, and IL-1Ra deletion on antibody production in our study offers insights into the dual role of IL-1β in promoting immune responses against pathogens while preventing autoimmunity.

Lupus is a systemic disease affecting mainly young women and can affect most organs. Biologically, this disease is characterized by the presence of antinuclear autoantibodies, in particular anti-native DNA and anti-RNP antibodies (27). Some manifestations such as renal involvement, are considered serious as they can be life-threatening. The injection of pristane in mice mimics lupus renal damage with the presence of glomerular immune complex deposits at 6 months (22, 28–32) as well as the development of anti-DNA autoantibodies in BALB/c mice and anti-RNP antibodies in C57BL/6 mice as early as 1 month after the injection (22). In this model, we could clearly see an impact of IL-1R1, IL-1R2, and IL-1Ra deletion on anti-RNP antibodies, but not on anti-native DNA antibodies; these changes are in line with the observation made after OVA immunization: the absence of an IL-1R1-mediated Tfr activation led to increased anti-RNP autoantibodies production, and the excess of IL-1R1-mediated activation led to the complete blockage of anti-RNP antibody production. This confirms an IL-1R control of Tfr suppression of antibody production for anti-RNP autoantibodies. The fact that this same regulation does not apply to the production of anti-DNA antibodies is puzzling. It may indicate an antigen-dependent regulation, may be related to the antigen abundance or affinity for antibodies. In any case, our results show that the immune complex deposition is correlated to the anti-DNA antibodies levels, but not to the anti-RNP levels. This is in line with the notion that in humans there is no clear correlation between autoantibody production and disease severity.

Recent findings by Griffith et al. in Nature Immunology (2023) demonstrated that IL-33 induces Treg cells in the lung to produce IL-1Ra. Given that both IL-33 and IL-1 signal through MyD88, this prompts the intriguing possibility that MyD88 is controlling a common pathway for IL-1Ra expression in these cells. In this line, it would be interesting to investigate the presence of the IL-33 receptor ST2 on Tfr cells.

Taken together, our results identify an overlooked role for IL-1 in the regulation of the GC reaction. While IL-1 can activate both Tfh and Tfr cells, the latter can only be activated at higher concentrations of IL-1 due to their concomitant expression of IL-1R2 and IL-1Ra. As IL-1 production progressively increases in lymph nodes after immunization, a Tfh mediated control of antibody production can occur before a suppression by Tfr, which activation occurs later. Our results warrant further mechanistic studies of this regulation. Future studies will be pivotal in further unraveling the complex interactions between IL-1β and follicular helper and regulatory T cells, offering new avenues for therapeutic interventions.

## Data availability statement

The raw data supporting the conclusions of this article will be made available by the authors, without undue reservation.

## Ethics statement

The animal studies were approved by Animal committee of the Centre d’Expérimentation Fonctionnelle animal facility (Paris, France) under specific pathogen–free conditions in agreement with current European legislation on animal care, housing, and scientific experimentation (agreement number A751315). The studies were conducted in accordance with the local legislation and institutional requirements. Written informed consent was obtained from the owners for the participation of their animals in this study.

## Author contributions

AB: Investigation, Data curation, Formal analysis, Methodology, Writing – original draft, Writing – review & editing. PE: Investigation, Writing – review & editing. GF: Formal analysis, Investigation, Methodology, Writing – review & editing. HV: Data curation, Formal analysis, Investigation, Writing – review & editing. RV: Data curation, Formal analysis, Software, Writing – review & editing. BG: Investigation, Writing – review & editing. BB: Writing – review & editing. IB: Formal analysis, Writing – review & editing. NT: Data curation, Formal analysis, Software, Writing – review & editing. SG: Conceptualization, Investigation, Methodology, Supervision, Validation, Writing – original draft, Writing – review & editing. DK: Conceptualization, Funding acquisition, Investigation, Methodology, Resources, Supervision, Validation, Visualization, Writing – original draft, Writing – review & editing.

## Supplementary material

Supplementary Figure 1 Phenotype of KO mice. **(A)** Relative expression of IL-1R1 in Tfol (CD4^+^CD19^-^PD1^+^CXCR5^+^), Tfh (CD4^+^Foxp3^-^CD19^-^PD1^+^CXCR5^+^) and Tfr (CD4^+^Foxp3^+^CD19^-^PD1^+^CXCR5^+^) cells from Foxp3^GFP^CD4^cre^IL-1R1^lox^ (green) and Foxp3^GFP^ (grey) mice by RT-PCR. IL-1R1 expression is evaluated as the relative expression corresponding to the IL-1R1 expression level of a cell population compared to its relative level in control mice. **(B)** Relative expression of IL-1R1 in Tfh and Tfr cells of Foxp3^creYFP^IL-1R1^lox^ (blue) and Foxp3^YFP^ (grey) mice by RT-PCR. IL-1R1 expression is assessed as previously mentioned. **(C)** Quantification of IL-1R2 expression on Tfr, Tfh, Treg (gated as CD4^+^Foxp3^+^CD19^-^PD1^-^CXCR5^-^) and Teff (gated as CD4^+^Foxp3^-^CD19^-^PD1^-^CXCR5^-^) cells from Foxp3^GFP^ control mice. **(D)** Quantification of IL-1R2 expression on Tfr, Treg and Breg cells (CD4^-^CD19^+^Foxp3^+^) from Foxp3^creYFP^IL-1R2^lox^ and Foxp3^creYFP^ mice. **(E)** Blood IL-1Ra expression in IL-1Ra^-/-^ (purple) and Foxp3^GFP^ (grey) mice after PBS or OVA-Alum immunization. *P < 0.05, **P < 0.01, ***P < 0.005, Mann-Whitney U test.

Supplementary Figure 2 Gating strategy. Representative gating B and follicular T cells before **(A)** and after **(B)** immunization.

Supplementary Figure 3 Quantification of Treg 28 days after immunization. Proportion of Treg and Treg/Tconv ratio 28 days after intraperitoneal immunization with OVA-Alum in CD4creIL-1R1lox (green triangle), Foxp3creYFPIL-1R1lox (blue triangle), Foxp3creYFPIL-1R2lox (red square), IL-1Ra-/- (purple diamond) and control mice (Foxp3creYFP, Foxp3GFP or C57BL/6J) mice (grey circle).
