# Supplementary figures and images for: Interleukin-1 regulates follicular T cells during the germinal center reaction
Source: Front Immunol. 2024 May 24;15:1393096. doi: 10.3389/fimmu.2024.1393096 (PMC11157057; doi:10.3389/fimmu.2024.1393096)

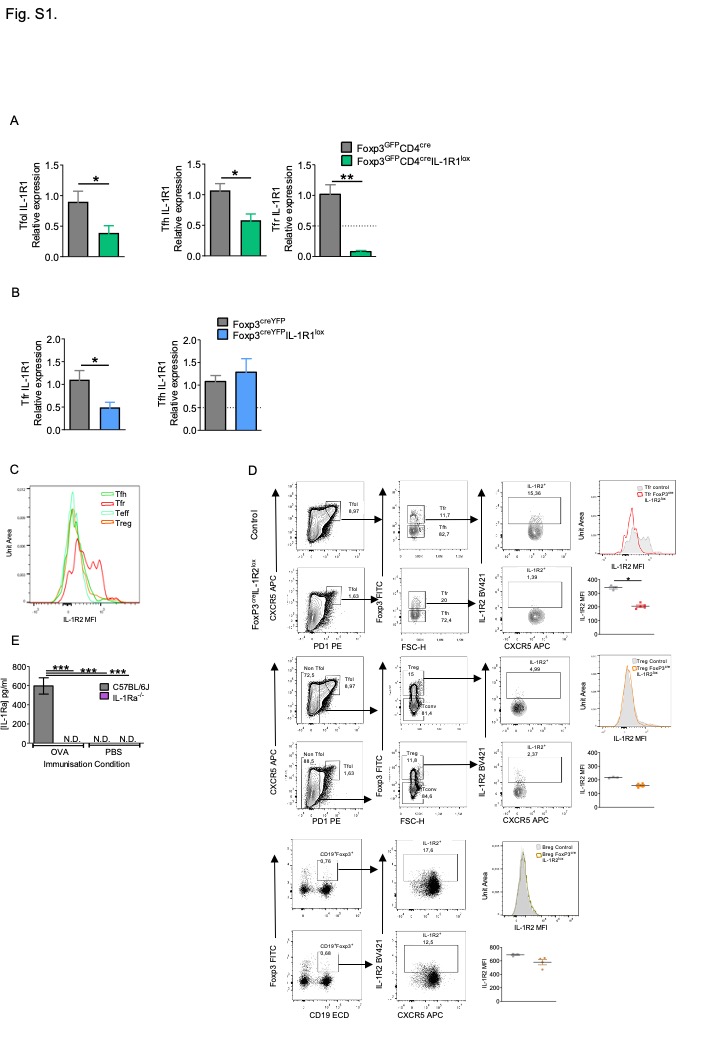

Supplement: Supplementary Figure 1 — Phenotype of KO mice. (A) Relative expression of IL-1R1 in Tfol (CD4+CD19-PD1+CXCR5+), Tfh (CD4+Foxp3-CD19-PD1+CXCR5+) and Tfr (CD4+Foxp3+CD19-PD1+CXCR5+) cells from Foxp3GFPCD4creIL-1R1lox (green) and Foxp3GFP (grey) mice by RT-PCR. IL-1R1 expression is evaluated as the relative expression corresponding to the IL-1R1 expression level of a cell population compared to its relative level in control mice. (B) Relative expression of IL-1R1 in Tfh and Tfr cells of Foxp3creYFPIL-1R1lox (blue) and Foxp3YFP (grey) mice by RT-PCR. IL-1R1 expression is assessed as previously mentioned. (C) Quantification of IL-1R2 expression on Tfr, Tfh, Treg (gated as CD4+Foxp3+CD19-PD1-CXCR5-) and Teff (gated as CD4+Foxp3-CD19-PD1-CXCR5-) cells from Foxp3GFP control mice. (D) Quantification of IL-1R2 expression on Tfr, Treg and Breg cells (CD4-CD19+Foxp3+) from Foxp3creYFPIL-1R2lox and Foxp3creYFP mice. (E) Blood IL-1Ra expression in IL-1Ra-/- (purple) and Foxp3GFP (grey) mice after PBS or OVA-Alum immunization. *P < 0.05, **P < 0.01, ***P < 0.005, Mann-Whitney U test. [file Image_1.jpeg]

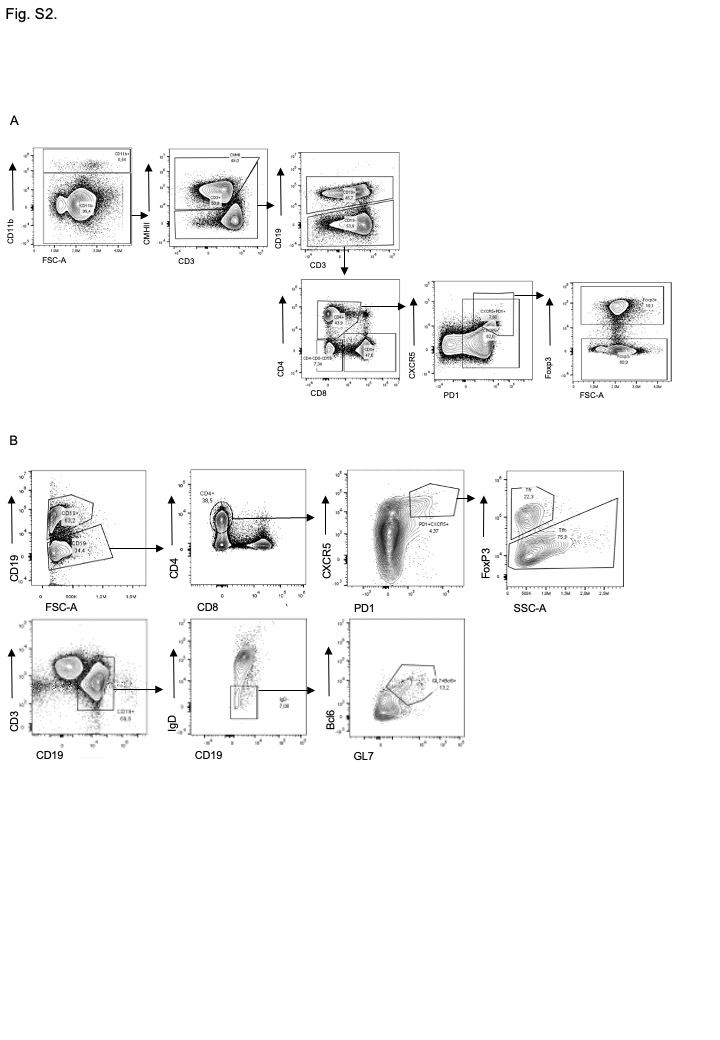

Supplement: Supplementary Figure 2 — Gating strategy. Representative gating B and follicular T cells before (A) and after (B) immunization. [file Image_2.jpeg]

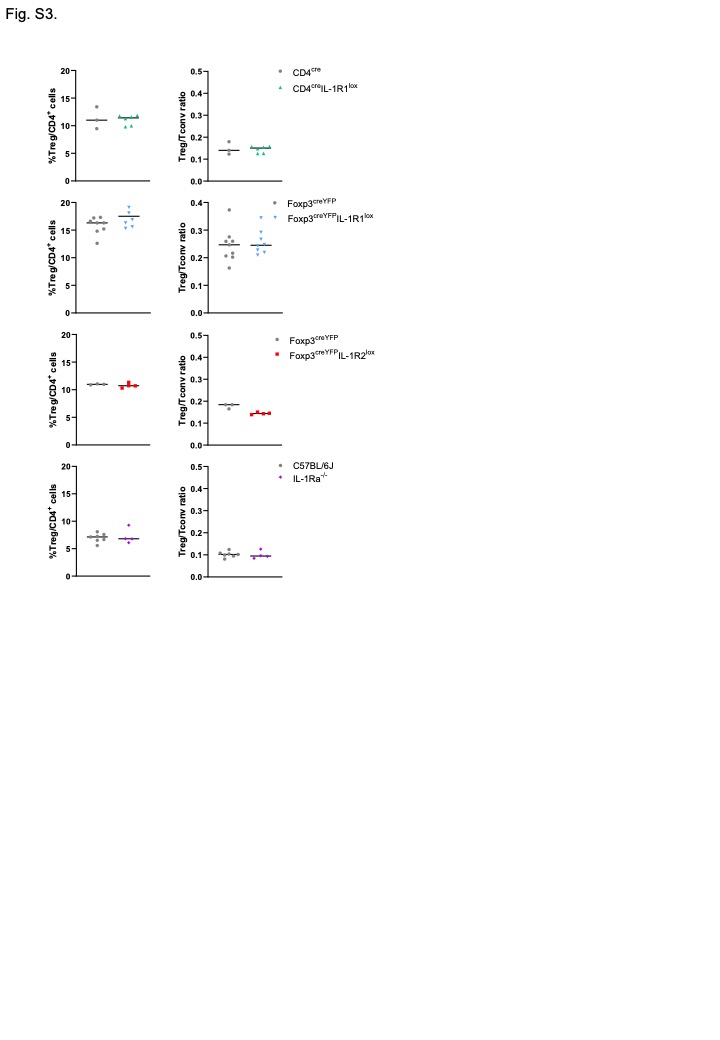

Supplement: Supplementary Figure 3 — Quantification of Treg 28 days after immunization. Proportion of Treg and Treg/Tconv ratio 28 days after intraperitoneal immunization with OVA-Alum in CD4creIL-1R1lox (green triangle), Foxp3creYFPIL-1R1lox (blue triangle), Foxp3creYFPIL-1R2lox (red square), IL-1Ra-/- (purple diamond) and control mice (Foxp3creYFP, Foxp3GFP or C57BL/6J) mice (grey circle). [file Image_3.jpeg]
